# Medullary Cancer of the Jaw in a Child

**Published:** 1868-12

**Authors:** Christopher Heath


### ARTICLE VIII.

### *Medullary Cancer of the Jaw in a Child.*

TWO SUCCESSFUL OPERATIONS; RETURN OF THE DISEASE; DEATH.

By Christopher Heath, F.R.C.S.,

The following case is of considerable interest, as illustrating the occurrence of undoubted medullary disease in a member of a large and healthy family with no history of cancer. The rapidity of the first growth in the lower jaw was such that the patient must shortly have sunk had the disease been allowed to go on. The operation secured an apparent restoration to health for exactly six weeks, when the disease reappeared, and in four days made such extraordinarily rapid progress that the patient's life was again in jeopardy. A second operation was followed by perfect recovery, and good health for ten weeks, when the disease again showed itself on both sides of the face, and, being now beyond surgical control proved rapidly fatal.

There can be no question that the poor child's life was prolonged for five months by surgical interference, as was gratefully acknowledged by the parents; and as patients ordinarily make good recoveries from even severe operations upon the jaws, it is to be regretted that cases are frequently allowed to pass into a hopeless state before active treatment, which offers the only chance for them, is undertaken.

Miss M. R——, aged five, was sent to me by Mr. Edward Randall, of Finsbury-square, on Sept. 9th, 1867, with a tumor of the lower jaw. She is the tenth of a family of eleven healthy children, and her parents are strong and robust. She is fat and well-nourished, though thinner than she was, and had perfect health until the last week in July (seven weeks before,) when her mother noticed that the second temporary molar tooth on the right side was raised above the others, and the gums looked swollen. Her mother took out the tooth, which was quite loose; but the swelling increased, and the first permanent molar became loose, and was extracted by Mr. Cole of Ipswich. She was under the care of Mr. Mumford of Ipswich, who used nitrate of silver lotion without benefit, since the growth continued to increase rapidly, so that she has been unable to eat solid food for a fortnight.

The whole of the lower jaw on the right side was considerably enlarged, and on opening the mouth, a large, irregular reddish mass was seen filling up all the cheek on the right side, the extent of which it was impossible to define. The tumor had a semi-elastic feel, and there were apparently no enlarged glands. There could be no question as to the propriety of, and necessity for, immediate operative interference, which I arranged to undertake on the following day.

On September 10th, 1867, the patient being under the influence of chloroform, I got my finger into the mouth, and then ascertained that the jaw was completely involved in the tumor, the elastic feeling being communicated through the bone. I divided the lower lip in the median line, and carried the incision round the border of the tumor to the level of the lobule of the ear. I then dissected back the flaps, and having divided the facial artery, tied it. Having extracted a loose tooth, I then sawed through the jaw immediately to the right of the symphisis, and detached the tissue on the inner side. On making traction, the tumor came away, leaving a rough, irregular piece of the jaw and a small portion of the tumor behind. These I subsequently extracted, including the condyle and coronoid process, which latter broke off and was removed separately, The internal maxillary artery was not wounded, and there was no great hæmorrhage, four ligatures being applied and cut short. The lip was brought together with two hare-lip pins, and the remainder of the wound closed by wire sutures, a silk suture being put in the red of the lip. Collodion was painted over all. No further dressing was applied. The child rallied, and took some brandy-and-water. In the afternoon she was quite comfortable, and the pulse was good. There was a little oozing from the wound. In the evening she had had some sleep, and had taken a little soup. She drank water frequently. There was no bleeding. The tumor proved to be of soft consistence, and had destroyed all the body of the jaw and a portion of the ramus; the condyle, coronoid process, and upper portion of the ramus being healthy. The point of section of the bone was healthy, and c[illegible]ose to it were the canine and first temporary molar. In the upper and posterior part of the growth was the crown of the second permanent molar, carried quite out of position. To the naked eye the tumor presented a loose fibroid appearance. Mr. Bruce kindly examined a portion microscopically for me, and reported numerous fibres, with here and there a development of cells, seemingly [illegible]edulla

September 11th.—She had had a comfortable night. The mouth syringed out with Condy's fluid three times. The child took some milk and soup, and was quite comfortable all day.

13th.—Child quite comfortable and happy, and takes liquid food well. I removed hare-lip pins.

14th.—I removed the sutures. The wound was healed except at the junction of the vertical with the horizontal incision, where there is a minute opening. The patient to be dressed and get up to-morrow.

17th.—Very comfortable and happy. The child eats bread-and-butter easily, and talks quite intelligibly. The interior of the mouth appears to be nearly healed. The left half of the jaw is thrown a little towards the median line.

18th—She went out in a perambulator.

23rd.—She went home to the country quite well, with the exception of one spot at the angle of the cicatrix, which still discharged slightly.

October 21st.—I heard that she was quite well.

26th—The child was brought to town on account of a return of the growth. The mother says she first noticed something wrong on the 22nd, when there was a small lump in the mouth. This grew very rapidly, and Mr. Mumford advised her coming up at once.

I found a mass within the mouth on the right side, nearly as large, and of precisely the same appearance, as the former growth. It involved a portion of the jaw left, and extended to the canine tooth on the left side, the incisors being loose. The cicatrix was sound except at the junction of the vertical with the horizontal incision, where the skin was ulcerated and there was a large fungous protrusion of the size of a cherry. I explained the serious nature of the case to the parents, and said that an immediate operation was the only hope, as, if left, the growth would rapidly fungate and destroy the child, and they consented to the operation proposed.

On the 27th October, 1867, I divided the lip and opened up the old cicatrix to a great extent, surrounding, however, the portion involved in the fungus. I then dissected back the flap, and found the growth extended to what would have been half way up the ramus. I isolated it, and then dissected back the left half of the lip. I next removed the first molar, and sawed the jaw close in front of the second molar. Having put a string in the tongue for safety, I then divided the sublingual muscles, and got the growth and piece of jaw away entire. Two or three large vessels were tied, principally under the tongue, and a few small ones. I washed the entire wound carefully with a solution of chloride of zinc (forty grains to the ounce,) brought the lip together with two hare-lip pins, and the remainder of the wound with sutures, and then fastened the string attached to the tongue to the upper pin in the lip. The child bore the operation very well. In the evening she was cold and restless, but rallied with the use of hot bottles and a little brandy,

A section of the tumor showed a beautiful specimen of soft cancer. Both tumors are now in the musuem of the College of Surgeons.

October. 28th—Patient had had a good night, and was asleep when I saw her at nine o'clock, and warm and comfortable. She passed a quiet day, taking a good deal of milk and a little wine. She was a little distressed by the ligature in the tongue.

29th.—She was comfortable, and took liquid food pretty well. Ordered her mouth to be syringed out.

30th.—Had passed a quiet night. I cut the thread in the tongue, and she was then able to move it about pretty freely. There was no difficulty of respiration. The wound was looking healthy, but the upper part of the old cicatrix was inflamed.

31st.—I removed the hare-lip pins and three of the stitches leaving those near the angle of the wound for the present. A little pus was pent up in the upper part of the old cicatrix, which I evacuated.

November 1st.—I removed the remaining stitches. The wound was healing well, except at the point where the skin was implicated and removed, and there it gaped.

3rd.—The child was up and dressed. She was able to close her lips and move her tongue very satisfactory. She takes her food fairly, and has sucked a chicken-bone

She continued to improve rapidly, and by the 10th, when she returned to the country, the wound was perfectly healed with the exception of a small spot where the portion of skin had been removed. She had perfect control over her tongue and lips, and could move the tissues of the chin very satisfactorily. There was no appearance of any return of the growth at this date.

December 16th.—I heard from the father that the child was perfectly well, and that there was no appearance of any return of the growth.

On the 8th ef January, 1868, I heard from Mr. Mumford that the disease had reappeared at the symphysis, and also in the masseteric region on both sides, there being loss of appetite, exhaustion, and general irritability of system. The poor little patient lingered for a month, and died on February 9th, just five months after I first saw her.—*Lancet.*

